# Lessons Learned from Enhancing Sentinel Surveillance for Cholera in Post-Earthquake Nepal in 2016

**DOI:** 10.4269/ajtmh.17-1008

**Published:** 2019-01-14

**Authors:** Kazutaka Sekine, Mellisa Roskosky

**Affiliations:** 1 UNICEF Sierra Leone, Freetown, Sierra Leone;; 2 Johns Hopkins University, Baltimore, Maryland

## Abstract

A major earthquake in 2015 that struck Nepal created a favorable environment for the rapid spread of infectious diseases. In anticipation of a cholera outbreak in 2016, UNICEF, Johns Hopkins University, and the Group for Technical Assistance, Nepal, collaborated to assist the government of Nepal to strengthen early warning surveillance, laboratory-based diagnosis, and field investigation. This article outlines the challenges and lessons learned in cholera prevention and control based on the authors’ experiences in 2016. Priorities for the future plan should include sustaining the enhanced surveillance system for acute gastroenteritis and cholera, rolling out a rapid diagnostic test, and ensuring rapid and systematic epidemiological investigation and environmental testing.

## INTRODUCTION

On April 25, 2015, a devastating earthquake struck Nepal. It was estimated that nearly 9,000 people were killed and more than 22,000 people were injured. Before the earthquake, cholera was considered endemic in Nepal, and cholera outbreaks posed a major public health threat, especially during the monsoon season as repeatedly reported internationally between 1958 and 2015.^1–11^ The disaster heightened the potential for an outbreak as it brought about extensive destruction of water supply infrastructure and sanitation facilities and collapsed health facility infrastructure.^12^ It also caused massive population displacement, creating a favorable environment for the rapid spread of infectious diseases. In 2015, after the earthquake, 76 acute watery diarrhea (AWD) cases were confirmed through bacterial culture as being caused by *Vibrio cholerae* and were reported in Kathmandu, one of the most affected districts.^11^

In 2016, the first cholera case was confirmed on June 30. A total of 169 cases and no deaths were reported that year. Of those cases, 150 were detected within the Kathmandu valley. In anticipation of a cholera outbreak in 2016, UNICEF, Johns Hopkins University (Baltimore, MD), and the Group for Technical Assistance (Kathmandu, Nepal), partnered to support the government of Nepal with the objective of strengthening capacity for disease surveillance, laboratory-based diagnosis, and epidemiological field investigation. This article discusses the challenges, lessons learned, and recommendations that were derived from the authors’ experiences in cholera prevention and response in 2016. The insights presented in the article aim to contribute to the scarce literature discussing cholera surveillance and diagnosis from a pragmatic perspective.^13–15^

## SURVEILLANCE FOR AWD AND CHOLERA

In 2016, with the support of its partners, the Epidemiology and Disease Control Division (EDCD) of the Ministry of Health (MoH) established a sentinel surveillance system specifically for AWD and cholera by setting up a network of 15 designated hospitals in the Kathmandu valley. Twelve of these sentinel surveillance sites were selected on the basis that these public hospitals represented large populations of different socioeconomic groups within different catchment areas and had medical staff trained to diagnose and treat patients with cholera. A standard case definition was used and suspected cases were defined as patients aged 1 year or more presenting with AWD, with or without vomiting. A case was considered confirmed when *V. cholerae* was isolated from their stool in bacterial culture. In addition, patients with a positive rapid diagnostic test (RDT) or any death resulting from AWD were defined as probable cases. As the number of cases began to rise, four additional private hospitals located in hotspot areas were temporarily added to the surveillance network as a measure to expand its reach to those seeking treatment at these hospitals. Inclusion of the private hospitals in the government’s surveillance was attributable to the leadership of the EDCD and the flexibility of the participating hospitals. The initiative included orientation to emergency doctors, laboratory technicians, and medical recorders on identification, recording, and reporting of suspected cases, provision of RDT kits used to trigger response activities (not for case confirmation), Cary-Blair media for transport of all samples to the National Public Health Laboratory (NPHL) for culture confirmation and serotyping, and capacity building for the use of these tools for diagnosis of *V. cholerae* infection. This sentinel surveillance improved the timeliness, completeness, and accuracy of alerts of suspected cases sent to the EDCD. The surveillance also generated the data necessary for situation reports that health authorities used for monitoring of the disease spread and planning of interventions.

Despite these efforts, the sentinel surveillance system for AWD and cholera in Nepal faced several limitations and challenges. First, despite the introduction of an incentive-based scheme, several sites failed to send daily reports on suspected cases to the EDCD because of an apparent lack of their engagement and motivation. Second, human resources within the EDCD for aggregating, analyzing, visualizing, and interpreting surveillance data remained understaffed and dependent on partners’ assistance. Third, a major limitation of the system lied in the difficulty of capturing cases outside of the Kathmandu valley. Detection of few cases outside the valley may be linked to absent or weak surveillance in those areas rather than the absence of the disease. Thus, the true burden of cholera may be underestimated, especially in remote areas.

Feasibility and practicality of the sentinel surveillance system within the Kathmandu valley were demonstrated. In the absence of a more agile and reliable system in Nepal, it remains crucial for early detection and timely response to cholera outbreaks. This highlights the need for the government and its partners to intensify their efforts to maintain and strengthen the surveillance system for AWD and cholera, as enshrined in the 2017 national preparedness and response plan for acute gastroenteritis and cholera outbreaks. It is important that the enhanced surveillance system eventually becomes integrated with the country’s current Early Warning and Reporting System.

## LABORATORY-BASED DIAGNOSIS

In accordance with national policy, confirmation of all suspected cholera cases in 2016 was conducted by bacterial culture^16^ at the NPHL, the top reference laboratory in Nepal, which resulted in overutilization of its capacity. A shortage of supplies and microbiologists with expertise in bacteriology posed a challenge in handling the large inflow of specimens for laboratory confirmation. Many district and regional hospital laboratories in Nepal are not capable of performing culture confirmation for the isolation and detection of *V. cholerae* due in large part to the lack of necessary laboratory supplies. Furthermore, the transportation of fresh stool samples required for culture confirmation from suspected cholera patients living outside the Kathmandu valley was challenging because of the lack of a pre-arranged transportation system.

We recommend that the MoH assess and strengthen laboratory culture capacity at both the regional and district levels to decentralize laboratory-based diagnosis. Once an outbreak has been declared, there is no need to confirm all suspected cholera cases by culture. Confirmatory testing for all suspected cases at the NPHL would overload the laboratory and lead to a delay in the monitoring of the outbreak. During outbreaks, the role of the NPHL should be limited to quality control of bacterial culture performed at the hospital or district level, as confirmation by bacterial culture is unlikely to influence the treatment of cholera cases.^17^ Even outside of the outbreak period, quality control by the NPHL should be maintained on a regular basis to monitor and strengthen the diagnostic capacities of decentralized laboratories. Increased resources for the procurement of laboratory supplies (e.g., media, reagents, and antisera) and the improved management of these supplies are required to ensure the uninterrupted availability of these essential commodities when and where they are needed. Furthermore, it is essential to establish a routine transportation system for transferring specimens to the NPHL and new methods of preserving samples for later confirmation when transporting fresh samples is impossible.

The introduction of a simple dipstick test for rapid diagnosis has enabled the sentinel surveillance to be a feasible and efficient way to detect cases rapidly. Our experience shows that the sensitivity and specificity of the dipstick test for the detection of *V. cholerae* O1 and O139 were 90% and 70%, respectively, when compared with bacterial culture (*n* = 194). Although RDTs vary greatly in quality,^18^ they require minimal technical skill and less time, and the RDT kits can be stored at room temperature allowing easy transport.^19^ They can be used to identify suspected cases rapidly in areas where laboratory facilities are not available or are inadequate. Ensuring the availability of RDTs and expanding rapid diagnosis across the country may be extremely useful to identify and respond to cholera outbreaks promptly.

## FIELD INVESTIGATION

Field investigation of households from which a cholera case was detected was conducted in a timely and standardized manner to discover the cause and/or source of the outbreak and carry out infection control measures. Field investigation provided opportunities to investigate the characteristics of the outbreak in terms of time, place, and person and to develop strategies for preventing future outbreaks. During the household visits, an epidemiological investigation was carried out using a standard questionnaire to collect information regarding the demographics of affected households, history of diarrhea among household members, food consumption, travel history, sources of drinking water, water treatment, sanitation conditions, and hygiene practices. In addition, water samples were collected from primary and secondary sources of drinking water for the isolation and identification of *V. cholerae* O1 and O139, and testing for fecal coliforms and residual chlorine. The bacterial culture at NPHL was used to detect *V. cholerae* from environmental water samples.^20^ *Vibrio cholerae* was isolated from samples of stored water. Households were notified if *V. cholerae* was isolated from their drinking water, and point-of-use water treatment products, soaps, and education on clean collection and storage practices were provided. Systematic use of a geographic information system for processing, analyzing, and visualizing spatial data was instrumental in investigating the geographic distribution of the disease, and for targeting interventions and follow-up visits.

A major challenge encountered during the field investigation was difficulty in tracking the patient’s family because of a lack of contact details recorded at the hospital or early discharge. Water sample testing added to the burden on the NPHL for laboratory testing. The lack of resources within the government for sustaining rapid, systematic, epidemiological investigation and environmental testing remains a critical challenge. These investigations provided valuable information in terms of transmission patterns and risk factors. We propose that rapid response teams at the district public health office conduct field investigations to ensure its sustainability.

## CONCLUSION

This article highlighted the challenges, lessons learned, and recommendations based on the authors’ experiences in cholera prevention and response in 2016. It provides practical insights into the gaps and a way forward in sentinel surveillance, laboratory-based diagnosis, and field investigation. The government of Nepal has taken much-needed steps to control cholera through the endorsement of the country’s first national cholera control plan, launched in 2017. However, there needs to be a high-level of political commitment to implement that national preparedness and response plan to end cholera as a threat to public health. Continued efforts are needed to develop effective and sustainable surveillance and laboratory diagnosis to rapidly detect and confirm cholera outbreaks.
